# A Diagnostic Challenge: A Case of Disseminated Nocardiosis Presenting With Generalized Lymphadenopathy in a Patient With Interleukin-12 Deficiency

**DOI:** 10.7759/cureus.62396

**Published:** 2024-06-14

**Authors:** Hussam Mitwalli, Yazeed Alekrish, Faris Nafisah, Abdullah Alkhamshi

**Affiliations:** 1 Division of Rheumatology, Department of Medicine, College of Medicine, King Saud University, Riyadh, SAU; 2 Department of Medicine, College of Medicine, King Saud University, Riyadh, SAU

**Keywords:** interleukin-12, nocardia farcinica, nocardia, adolescent medicine, disseminated nocardia

## Abstract

Deficiency in interleukin-12 (IL-12) can result in susceptibility to opportunistic infection, with IL-12 deficiency being a rare genetic cause. *Nocardia farcinica* is a gram-positive aerobic actinomycete that can cause disseminated and potentially lethal nocardiosis in immunocompromised patients. This report describes a 16-year-old male adolescent with IL-12 deficiency presenting with generalized lymphadenopathy due to disseminated *Nocardia farcinica.*

The subject of our study is a male adolescent who exhibited clinical manifestations consistent with cholestasis. He underwent extensive workup for malignancy, suspecting cholangiocarcinoma initially. The workup turned out unremarkable, and later during his hospital stay, he deteriorated and required intensive care unit (ICU) admission, as he developed superior vena cava (SVC) syndrome from massive enlargement of mediastinal and cervical lymph nodes. During the patient's admission, it was found that he had a deficiency of interleukin-12 (IL-12). Later on, a blood culture revealed the presence of *Nocardia farcinica *species. Subsequently, the patient was initiated and improved drastically on an empirical antibiotic regimen consisting of amikacin, co-trimoxazole, meropenem, and moxifloxacin. Following that, the susceptibility results came out, and he was switched to oral co-trimoxazole and oral moxifloxacin as he no longer required inpatient care.

This report highlights the importance of accurate diagnosis of causes of immunosuppression and early investigation, diagnosis, and management of potentially fatal opportunistic infections such as disseminated *Nocardia farcinica.*

## Introduction

*Nocardia* species are filamentous, aerobic, gram-positive, rod-shaped bacteria with weak acid-fast properties that belong to the actinomycetes group and specifically to the family of Mycobacteriaceae [1-3]. *Nocardia* sp. infections can be transmitted by ingestion or inhalation and could subsequently cause cutaneous nocardiosis, central nervous system (CNS) nocardiosis, pulmonary nocardiosis, or disseminated nocardiosis [4]. The cutaneous form is the most common form that occurs in immunocompetent individuals, often affecting those working in rural areas or involved in agricultural activities [5]. On the other hand, pulmonary nocardiosis is the most common form overall and typically occurs in immunocompromised patients, such as those with solid organ transplants, HIV infection, chronic corticosteroid use, active malignancy, or any defect in cellular immunity. Disseminated nocardiosis is defined as *Nocardia *infection affecting two non-contiguous sites [1,3-8].

Although the incidence of nocardiosis has been on the rise in the past decade, as reported in a Canadian study that the yearly incidence has risen from 0.33 to 0.87 cases per 100,000 individuals [9], it is still considered an uncommon cause of infection, with approximately 500-1000 cases reported each year in the United States [10] and 90-130 cases in Italy [11]. Nocardiosis is more prevalent in tropical regions of the world, such as India, Pakistan, Iran, Canada, and Spain [9-12].

The presentation of nocardiosis is variable and depends greatly on the organ involved, the most common of which is pulmonary involvement, which could present with the signs and symptoms of pneumonia. It could also lead to cavitary lesions, abscess formation, pleural effusion, or empyema. It could also disseminate to virtually any organ system in the body and develop manifestations related to that specific organ. The most common site of dissemination is the brain, as it is detected in around 44% of disseminated *Nocardia* infections, which could present as a slowly progressive mass lesion with neurologic findings related to the specific location of the abscess [8,12].

The diagnosis of nocardiosis requires a high index of suspicion, and it should be included in the differential diagnosis when the patient presents with features of pulmonary involvement along with brain involvement or multiple distant site involvement, especially with the history of being immunocompromised [12-14].

Any condition or factor that makes someone immunocompromised is considered a risk factor for *Nocardia* infection; however, it has been reported that specifically, conditions that impair cell-mediated immunity are associated with *Nocardia* infections, especially conditions causing impaired phagocyte function, such as interleukin-12 (IL-12) deficiency. IL-12 is a cytokine that plays an important role in mediating cellular immunity, as it facilitates the differentiation of naïve CD4+ T cells into Th1 cells, which in turn produce interferon-gamma (IFN-γ) [15,16].

This case report describes a 16-year-old adolescent male with IL-12 deficiency presenting with generalized lymphadenopathy due to disseminated *Nocardia farcinica*.

## Case presentation

A 16-year-old medically and surgically free male presented to the emergency department with a one-month history of upper abdominal pain that was primarily related to fatty foods. His family also observed a two-week history of yellowish discoloration of his skin. He also reported other symptoms, such as pale stool, dark urine, unintentional weight loss (from 7 to 10 kg in one month), night sweats (for two weeks), and two episodes of fever reaching as high as 38.5°C over 10 days. He had no history of pruritus, chest pain, dyspnea, urinary tract symptoms (other than a change in urine color), changes in bowel habits, recent contact with sick individuals, or recent travel. On examination, his heart rate (HR) was 83 beats per minute, blood pressure was 107/68 mmHg, respiratory rate was 18 per minute, and body temperature was 36.8°C. His abdominal examination was unremarkable, with no tenderness or guarding throughout the abdomen. His chest examination was also unremarkable with clear vesicular normal breathing sounds bilaterally.

**Figure 1:**
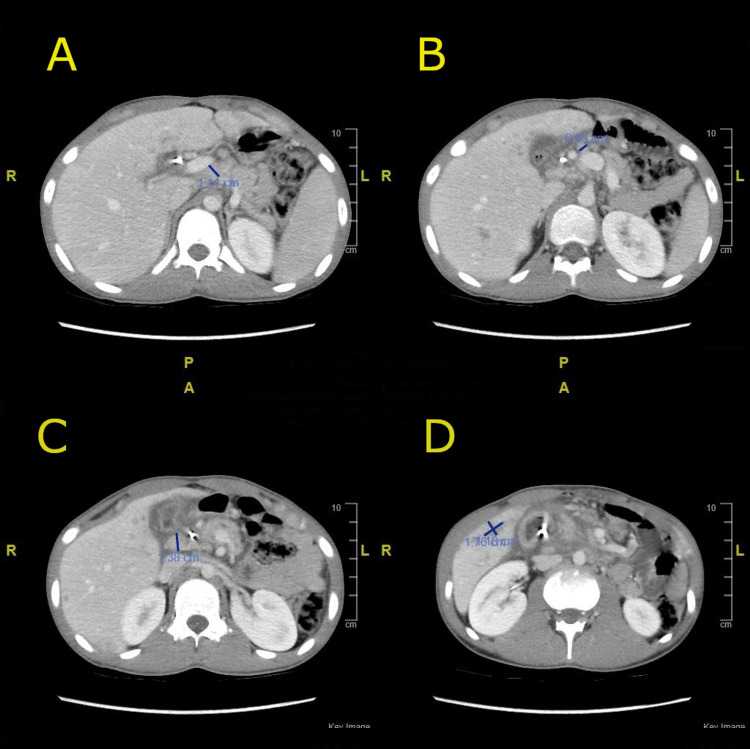
CT scan of the abdomen upon admission A: Axial CT image showing enlarged lymph node in the celiac region measuring 1.11 cm. B: Axial CT image showing enlarged lymph nodes in the para-aortic region measuring 0.63 cm. C: Axial CT image showing enlarged upper abdominal lymph nodes, the largest of which is in the porta hepatis, measuring 1.38 cm. D: Axial CT image showing multiple ill-defined focal hypodense hepatic lesions, the largest of which measures 1.8 × 1.2 cm. CT: computed tomography

Three days prior to presenting to our hospital, the patient sought treatment at a secondary hospital, where a computed tomography (CT) scan revealed moderate intrahepatic biliary duct dilation (more prominent in the left lobe), a mass effect on the left main portal vein, and multiple enlarged hepatic hilar lymph nodes (Figure 1).

At the time of admission, a routine hemogram revealed mild leukocytosis 12 × 10^9^/L (reference range: 4-11 × 10^9^/L) with eosinophilia, microcytic hypochromic anemia (hemoglobin: 112 g/L, reference range: 130-180 g/L), and thrombocytosis (platelet: 457 × 10^9^/L, reference range: 140-450 × 10^9^/L). His liver function tests (LFTs) were moderately deranged, with aspartate aminotransferase (AST) of 53.5 unit/L, alanine transaminase (ALT) of 55.2 unit/L (reference range: 0-40 unit/L), gamma-glutamyl transferase (GGT) of 118 unit/L (reference range: 8-61 unit/L), and alkaline phosphatase (ALP) of 314 unit/L (reference range: 82-331 unit/L).

The patient underwent a comprehensive and extensive malignancy workup in the following days. His cancer antigen 19-9 (CA 19-9) level was 55.4 kU/L (reference range: 0-27 kU/L). The results of investigations for infectious etiologies, such as tuberculosis (Quantiferon) and hepatitis B virus (HBV) serology, were negative. Sputum Ziehl-Neelsen (ZN) staining for acid-fast bacilli turned out negative as well. Magnetic resonance cholangiopancreatography (MRCP) revealed circumferential ductal wall thickening and polypoidal lesions of the common hepatic and bile ducts, raising concerns for cholangiocarcinoma. Afterward, endoscopic retrograde cholangiopancreatography (ERCP) was performed, and biopsies were obtained. However, all biopsies turned out unremarkable for malignancy.

**Figure 2:**
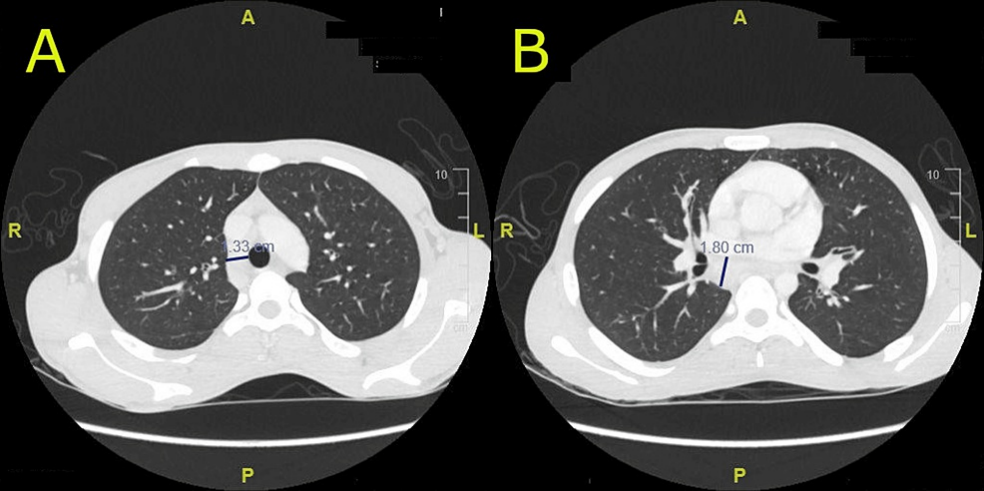
CT scan of the chest A: Axial CT image showing enlarged mediastinal lymph nodes, with the right paratracheal lymph node measuring 1.33 cm. B: Axial CT image showing enlarged mediastinal lymph nodes, the largest of which is in the subcarinal region measuring 1.8 cm. CT: computed tomography

During this time, no clear diagnosis was established. Ultrasounds of the groin and neck showed significant lymphadenopathy, and a CT scan of the chest, abdomen, and pelvis revealed many poorly defined hepatic lesions suggestive of hepatic microabscesses and multiple enlarged mediastinal lymph nodes (Figure 2). The patient underwent endoscopic ultrasound (EUS) and lymph node biopsy since multiple enlarged lymph nodes were identified. His lipase level was elevated at 707 U/L at that time for no clear reason. The lymph nodes of the pancreas and the ampulla of Vater were biopsied, revealing diffuse eosinophilic infiltration but were unremarkable for malignancy. A liver biopsy was done, which only revealed mild periportal chronic inflammation with minor periportal fibrosis (grade 1, stage 1, Batts-Ludwig system), and was unremarkable for malignancy.

**Figure 3:**
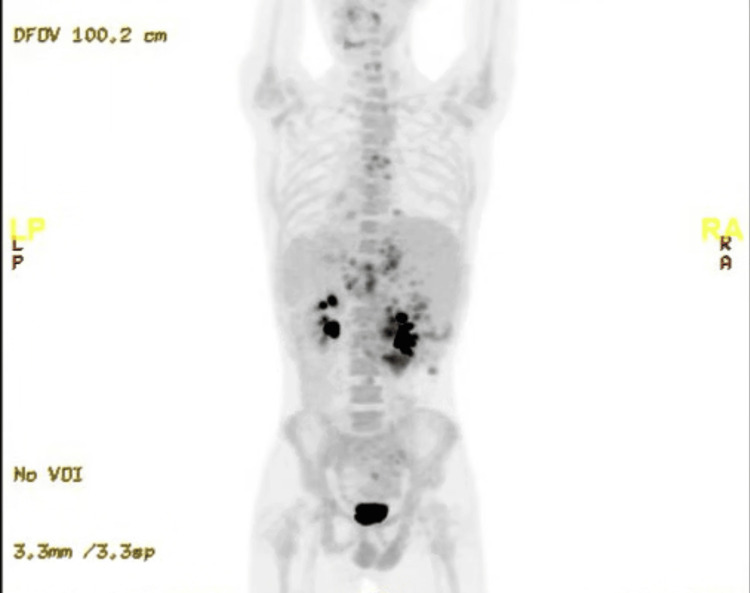
Whole-body PET scan The scan revealed multiple FDG-avid hepatic lesions with a maximum SUV of 6.7 in the right hepatic lobe; there was also intense FDG uptake in the duodenal wall (SUVmax: 8.7), suggestive of infectious, inflammatory, or disease involvement. The mediastinum displayed several hypermetabolic lymph nodes, including subcarinal (SUVmax: 5.7) and upper right paratracheal (SUVmax: 3.4) nodes. Additionally, diffuse homogeneous increased bone marrow FDG activity was noted. PET: positron emission tomography, FDG: fluorodeoxyglucose, SUV: standardized uptake value

The patient's positron emission tomography (PET) scan showed hypermetabolic hepatic lesions, mediastinal lymphadenopathy, hypermetabolic upper abdominal lymphadenopathy, and increased duodenal wall ^18^F-fluorodeoxyglucose (FDG) activity, as well as bone marrow FDG activity (Figure 3).

Lymphoma was considered since the patient started to develop generalized lymphadenopathy. However, all lymph node biopsies were negative. Bone marrow biopsy with cytogenetics and karyotyping and mediastinoscopy with subcarinal lymph node biopsy were unremarkable for malignancy. Additional workup included genetic testing where a homozygous pathogenic variant in the IL12B gene 5q33.3 was identified, confirming the diagnosis of IL-12 deficiency and autosomal recessive immunodeficiency type 29.

Although IL-12 deficiency was confirmed, no diagnosis for this acute illness was established yet. The patient's clinical condition deteriorated, as evidenced by the onset of productive cough, facial puffiness, fever (reaching 39.5°C), tachycardia (reaching 117 beats per minute), increased oxygen demands, and airway compromise. He was started on meropenem, vancomycin, and dexamethasone; his septic workup, including blood, urine, and sputum cultures, were all unremarkable. As a result of airway compromise due to severely enlarged lymph nodes, a central venous line was placed for hemodynamic monitoring, and the patient was intubated and admitted to the intensive care unit (ICU). A CT scan of the chest confirmed the presence of superior vena cava (SVC) syndrome as a result of the massive enlargement of mediastinal lymph nodes. In addition, the para-aortic and mesenteric lymph nodes were enlarged, resulting in the compression of the third part of the duodenum. A nasojejunal tube (NJT) was inserted to facilitate enteral nutrition during the patient's stay in the intensive care unit (ICU). Multiple cervical lymph nodes were significantly enlarged, and bilateral pleural effusion with atelectasis was also identified. Due to his hemodynamic instability, inotropic agents were administered to maintain an arterial mean pressure above 65 mmHg. His liver function tests exhibited a marked elevation, with AST levels reaching 1,130 unit/L (reference range: 0-40 unit/L) and ALT levels reaching 377 unit/L (reference range: 0-40 unit/L).

A point-of-care chest ultrasound detected pleural effusion with septation and bilateral loculation approximately four days later. To address this issue, bilateral chest tubes were subsequently inserted. Pleural fluid was sent for gram stain, bacterial culture, and ZN staining; however, they all turned out to be negative for any organisms. Bronchoscopy was performed, revealing the right mainstem bronchus narrowing due to mass effect. Multiple biopsies were taken during the bronchoscopy, all of which were unremarkable for malignancy. Doppler ultrasound showed that the patient developed acute deep vein thrombosis (DVT) affecting his left subclavian, axillary, and brachial veins. In response, enoxaparin therapy was initiated. Following the initiation of empirical antimicrobial therapy, the patient's condition significantly improved. He was extubated with success and no longer required supplemental oxygen. As a result, the NJT was removed, and he was transferred to the general ward.

Two days later, a new clinical issue emerged, manifested by a painful collection on the anterior chest wall, fever, and tachycardia. He was then diagnosed with an anterior chest wall abscess, for which incision and drainage were done, along with the antimicrobials he was already on. Five days after incubation, *Nocardia farcinica* isolates were detected in blood cultures using the BACT/ALERT® (bioMérieux, Marcy l'Etoile, France) system. The organism was subsequently identified using matrix-assisted laser desorption/ionization time-of-flight mass spectrometry (MALDI-TOF MS).

This discovery led to the diagnosis of disseminated nocardiosis. The patient's injectable antimicrobial regimen was changed to cotrimoxazole (240 mg TID), meropenem (1 g TID), moxifloxacin (400 mg OD), and amikacin (700 mg OD), and further susceptibility testing was sent. During this time, the patient was evaluated to rule out brain dissemination. A CT scan of the brain was unremarkable for any involvement of the brain. The patient underwent daily evaluation of his electrocardiogram (ECG) for any signs of QT prolongation. Additionally, amikacin levels were monitored weekly to detect and manage any potential toxicity.

During his stay, the patient received a total of 26 days of intravenous (IV) antibiotics. He demonstrated remarkable clinical improvement on these antimicrobials, the symptoms resolved, and the chest tubes were removed. Antimicrobial susceptibility testing revealed that *Nocardia farcinica *was sensitive to cotrimoxazole, moxifloxacin, imipenem, and amikacin. After the susceptibility results came out, he was discharged from the hospital and given oral cotrimoxazole (2 DS tablets, BID) and oral moxifloxacin (400 mg OD) as an outpatient regimen for one year. Concurrently, his anticoagulant medication was changed to oral rivaroxaban for a total duration of three months. The patient was then discharged after showing complete clinical resolution of his symptoms and continued following up in the clinic. He is currently doing well, with no issues, as he continues his long-term antimicrobial therapy for one year.

## Discussion

Nocardiosis is an infrequent cause of infection that is often not considered an initial diagnosis during a patient's presentation. However, it necessitates an increased level of suspicion due to its potential to impact several organs within the body and its tendency to affect many sites, particularly in individuals with impaired immune systems [1-3,6-8]. This case highlights the importance of rapid and appropriate identification of *Nocardia* infections in immunocompromised patients.

IL-12 deficiency is a rare inherited cytokine deficiency that can result from mutations in the IL12A or IL12B genes or in the genes responsible for IL-12 receptors [17]. The presence of IL-12 deficiency leads to abnormalities in the differentiation of naïve T cells into Th1 cells. The role of these cells is to produce interferon-gamma (IFN-γ), which is an essential cytokine in cell-mediated immunity that helps in activating macrophages, enhancing the elimination of intracellular pathogens. Therefore, a deficiency in IL-12 can result in increased susceptibility to infections, particularly from intracellular organisms. IL-12 also has other roles, as it has been found to enhance the cytotoxic activity of natural killer (NK) cells and cytotoxic T lymphocytes (CTLs) [18-20].

IL-12 deficiency was confirmed in our patient by genetic testing, which revealed a homozygous pathogenic variant in the IL12B gene 5q33.3. Although both our patient and his sibling had *Nocardia* infection, we could only hypothesize that the source of their infections was something in their environment. It is worth noting that the patient's family lives outside Riyadh, in a rural area in the northern region of Saudi Arabia.

In our case, disseminated nocardiosis infection was mimicking metastatic disease; this unusual presentation has been reported in the literature before, such as one case of disseminated nocardiosis caused by *Nocardia cyriacigeorgica*, which presented with the picture of metastatic malignancy and multiple lesions in the brain and lungs. However, the diagnosis was revised when bronchoalveolar lavage (BAL) cultures revealed the presence of *Nocardia* *cyriacigeorgica* [13]. This was also similar to another case, which presented with a high suspicion of lung cancer metastasis to the adrenal gland; however, wedge resection of the pulmonary nodules revealed *Nocardia* infection [21].

The management of disseminated nocardiosis usually requires a prolonged course of antimicrobial therapy. Current practice is to give antibiotics for several months, sometimes up to a year, to fully eradicate the infection. It is usually recommended to give a minimum of a six-month course of antibiotic therapy, with the first-line drug being trimethoprim-sulfamethoxazole (TMP-SMX) [5,12,19,22].

## Conclusions

This case had an unusual presentation mimicking malignancy, and after extensive workup and investigations, it was revealed that this patient had IL-12 deficiency that was complicated with disseminated nocardiosis caused by *Nocardia farcinica*. It also demonstrates the need for staying vigilant and having a higher index of suspicion when dealing with nocardiosis, as it is a fairly difficult diagnosis to make. This report highlights the importance of accurate diagnosis of causes of immunosuppression and early investigation, diagnosis, and management of potentially fatal opportunistic infections such as disseminated *Nocardia farcinica*. In addition, it highlights the need for better and available diagnostic methods for *Nocardia* species. Modern techniques for microorganism identification, such as polymerase chain reaction-restriction fragment length polymorphism (PCR-RFLP) amplification of a portion of the *Nocardia* 16S rRNA gene, are specific and accurate diagnostic methods. However, they mark a logistic difficulty with difficult availability in many hospitals, which was the case in our institution. Luckily, a single blood culture turned out to be positive in this patient, which confirmed the diagnosis.
